# Supplementary material for: Aging-Induced Changes in Cutibacterium acnes and Their Effects on Skin Elasticity and Wrinkle Formation
Source: Microorganisms. 2024 Oct 29;12(11):2179. doi: 10.3390/microorganisms12112179 (PMC11596587; doi:10.3390/microorganisms12112179)
Supplement: Supplementary file 1 [file microorganisms-12-02179-s001.zip › microorganisms-3265366-supplementary.pdf]

Supplementary materials

Aging-Induced Changes in *Cutibacterium acnes* and Their Effects on Skin Elasticity and Wrinkle Formation

YeonGyun Jung [1,†], Ikwhan Kim [2,†], Da-Ryung Jung [2], Ji Hoon Ha [3], Eun Kyung Lee [4], Jin Mo Kim [3], Jin Young Kim [3], Jun-Hwan Jang [5], Jun-Tae Bae [5], Jae-Ho Shin [2,6,7,*] and Yoon Soo Cho [4,*]

1 Burn Institute, Hangang Sacred Heart Hospital, Hallym University College of Medicine,
Seoul 07247, Republic of Korea; jyg1076@gmail.com (Y.J)

2 Department of Integrative Biology, Kyungpook National University, Daegu 41566, Republic of Korea; ikwhankim0926@gmail.com (I.K.); amugae1210@knu.ac.kr (D.-R.J.)

3 R&D Center, Kolmar Korea, Seoul 06800, Republic of Korea; jh_cos@kolmar.co.kr (J.H.H.); kimjinmo@kolmar.co.kr (J.M.K.); jinyoung_cos@kolmar.co.kr (J.Y.K.)

4 Department of Rehabilitation Medicine, Hangang Sacred Heart Hospital, Hallym University College of Medicine, Seoul 07247, Republic of Korea; eunlee0617@gmail.com

5 J2KBIO, Chungbuk 28104, Republic of Korea; jjh1117@j2kbio.com (J.-H.J.); jtbae@j2kbio.com (J.-T.B.)

6 Department of Applied Biosciences, Kyungpook National University, Daegu 41566, Republic of Korea

7 KNU NGS Core Facility, Kyungpook National University, Daegu 41566, Republic of Korea

* Correspondence: jhshin@knu.ac.kr (J.-H.S.); yschorm@hallym.ac.kr (Y.S.C.)¿ Tel.: +82-53-950-5716 (J.-H.S.); +82-02-2639-5739 (Y.S.C.)

† These authors contributed equally to this work.

Table S1. Spearman's correlation between participant characteristics and *Cutibacterium acnes* abundance

Variables	*Cutibacterium acnes*	
	r	p-value
TEWL, g/m²/h	0.033	0.801
pH	0.119	0.363
Sebum, μg sebum/cm²	0.177	0.176
Melanin, AU	0.068	0.606
Erythema, AU	0.007	0.959
Final distensibility (R0), mm	-0.004	0.975
Viscoelasticity (R6), %	-0.075	0.569

Abbreviations: TEWL, transepidermal water loss; AU, arbitrary unit.
